# Childhood vaccination coverage and socioeconomic inequalities in the Northern region of Brazil

**DOI:** 10.1590/1980-549720260027

**Published:** 2026-07-27

**Authors:** Eduarda de Paulo Costa, Luiz Fernando Costa Nascimento

**Affiliations:** I Universidade de São Paulo, Medical School – São Paulo (SP), Brazil.; II Universidade Estadual Paulista Júlio de Mesquita Filho, School of Engineering and Sciences – Guaratinguetá (SP), Brazil.

**Keywords:** Vaccine coverage, Spatial analysis, Health inequalities, Socioeconomic factors, Child health

## Abstract

**Objective:**

To evaluate the spatio-temporal variation of childhood immunobiological vaccine coverage in the municipalities of the Northern region of Brazil, comparing cross-sectional data from 2018 and 2022, and its association with socioeconomic and maternal variables.

**Methods:**

This is a comparative ecological study with coverage data for seven immunobiologicals in the municipalities of the Northern region for the years 2018 and 2022. Data were obtained from the Department of Informatics of the Unified Health System. Spatial analysis (global, bivariate, and local Moran’s Index) was used to verify the association between vaccine coverage and independent variables (GDP *per capita*, Brazilian Deprivation Index, maternal schooling, proportion of indigenous people, full-term births, and birth weight).

**Results:**

There was a widespread decline in vaccine coverage when comparing the two periods, except for the meningococcal C vaccine. Vaccination was positively associated with GDP *per capita* and maternal schooling, and negatively associated with the Deprivation Index, with these associations intensifying over the period. The spatial correlation between immunization and the proportion of the indigenous population, previously not significant, became strongly negative in 2022. Clusters of low immunization and socioeconomic vulnerability were identified mostly in the states of Pará and Amazonas, and clusters of high immunization and better indicators in Tocantins and Rondônia.

**Conclusion:**

The decline in childhood vaccination in the Northern region is a spatially structured phenomenon that reflects and deepens socioeconomic inequalities. The formation of vulnerability clusters, especially in areas near the Brazilian border, represents a risk for the re-emergence of vaccine-preventable diseases and demands territorially focused public health policies.

## INTRODUCTION

Vaccination is one of the most effective and cost-effective public health strategies for reducing infant mortality, preventing disease, and promoting healthy development in childhood. Since the implementation of large-scale vaccination strategies, such as the World Health Organization’s (WHO) Expanded Programme on Immunization, it is estimated that more than 154 million deaths have been averted worldwide, including 146 million among children under five years of age and 101 million among infants^1,2,3^.

In Brazil, the National Immunization Program, established by the Ministry of Health in 1973, provides a comprehensive vaccination schedule free of charge and is internationally recognized for its capacity to eradicate vaccine-preventable diseases, such as the eradication of wild poliovirus and interruption of rubella virus circulation, as well as for the significant reduction in cases and deaths from diseases such as diphtheria, tetanus, measles, and hepatitis B^4^.

However, since 2016, a decline in vaccination coverage (VC) has been observed across the country^4^. This trend intensified during the COVID-19 pandemic, which overwhelmed the health system, restricted mobility, and caused fear of exposure to the virus in health services, resulting in sharp declines in vaccination rates^5^. Regional heterogeneity is remarkable, with the North and Northeast regions showing the largest declines^6^, although the phenomenon is nationwide and even affects areas historically characterized by high coverage^7^.

Migration from neighboring countries, especially Venezuela and Guyana, has significantly impacted public health and VC in municipalities along Brazil’s northern border. Increased human mobility driven by humanitarian crises and economic activities, such as mining, is associated with a rise in imported cases of transmissible diseases, like malaria, viral hepatitis, and measles, as well as challenges in maintaining high VCs^8,9,10,11,12^.

Calculating VC at state or national levels can mask vulnerable areas by diluting spatial and social heterogeneities through aggregated averages. Without granular analysis, interventions may be poorly targeted, perpetuating inequalities and vulnerabilities^13,14,15,16,17^. One methodological alternative is spatial analysis at the municipal level, which allows mapping areas of epidemiological risk and facilitates the detection of underlying social determinants.

In this context, this study aims to evaluate the spatial and temporal variation of VC for immunobiologicals administered in childhood in municipalities of Brazil’s Northern region between 2018 and 2022, as well as its association with socioeconomic and maternal variables.

## METHODS

**Figure 1:**
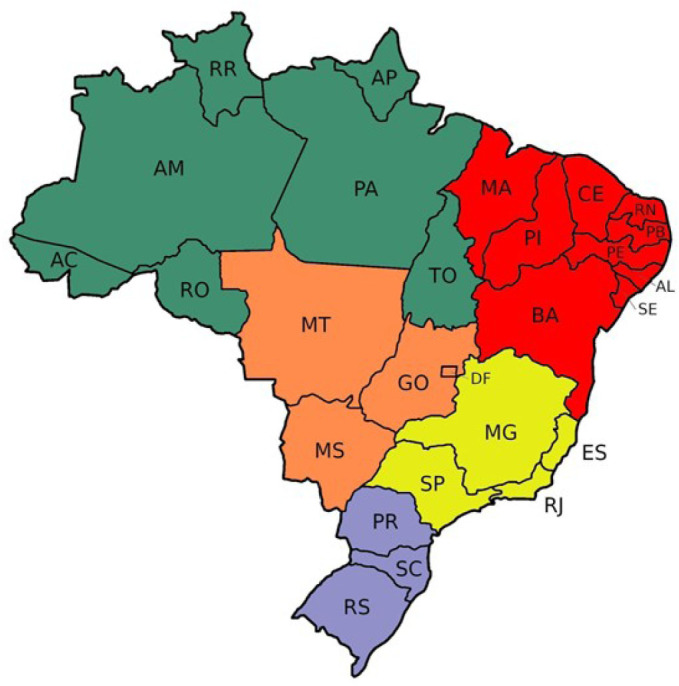
Political map of Brazil identifying the Northern Region and its constituent states: Acre (AC), Amapá (AP), Amazonas (AM), Pará (PA), Rondônia (RO), Roraima (RR) and Tocantins (TO).

This is an ecological study using VC data from 450 municipalities in the seven states of the Northern region (Figure 1)^18^ for the years 2018 and 2022, obtained from the Department of Informatics of Brazil’s Unified Health System (DATASUS)^19^.

The selected immunobiologicals comprised those recommended for childhood vaccination, namely: BCG (tuberculosis), pentavalent (diphtheria, tetanus, pertussis, hepatitis B, and *Haemophilus influenzae* type b), poliomyelitis, pneumococcal, rotavirus, meningococcal C, and measles-mumps-rubella (MMR).

VCs (dependent variable) were obtained as percentages by antigen for each municipality. Mean values were compared between 2018 and 2022. Subsequently, localities that did not reach the coverage levels recommended by the Ministry of Health were identified: 90% for BCG and rotavirus and 95% for the other immunobiologicals.

The socioeconomic indicators (independent variables) chosen were gross domestic product (GDP) *per capita* and the Brazilian Deprivation Index (Índice Brasileiro de Privação, IBP), as well as maternal variables such as education (eight or more years of schooling), race (proportion of Indigenous people among live births), low birth weight (<2,500 g), and gestational duration (term births), all expressed as percentage rates.

Created by researchers from Fiocruz Bahia and the University of Glasgow, the IBP measures material deprivation based on information about income, education, and housing conditions at the smallest territorial unit used by the Brazilian Institute of Geography and Statistics (IBGE), the census tract, which allows identification of pockets of poverty within municipalities. This index can take positive and negative values, with higher positive values indicating greater deprivation.

For the analysis of spatial dynamics, global Moran’s I statistics and their p-values were estimated to assess spatial autocorrelation among municipalities for each variable. Bivariate and local Moran’s I (LISA) indices were also calculated.

Moran’s I provides a measure of spatial association for a set of data on a scale from −1 to +1. Positive values (positive autocorrelation) indicate similarity between the values of the variables and their spatial locations; negative values (negative autocorrelation) indicate dissimilarity; and values near zero indicate a random spatial distribution. The global Moran’s I measures spatial autocorrelation for the entire dataset; for example, it assesses whether there is a global spatial pattern across the study area. The bivariate Moran’s I is used to assess spatial autocorrelation between two distinct variables, examining whether high (or low) values of one variable in a location are associated with high (or low) values of another variable in neighboring locations. LISA, in turn, indicates whether a locality and its neighbors have similar values (high–high or low–low clusters) or dissimilar values (high–low or low–high), enabling the identification of areas with distinct spatial patterns within the overall dataset.

For defining spatial neighbors, a first-order Queen contiguity weight matrix was used, row-standardized, considering as neighbors the cities that share common borders or vertices. This choice is justified by the extreme heterogeneity of municipal territorial areas in the Northern Region, which makes fixed-distance centroid-based matrices unfeasible, as they could create false isolation in extensive municipalities or excessive connections in denser areas. The Queen matrix was chosen because it increases topological connectivity among spatial units, reducing the impact of irregular municipal geometries and ensuring that all administrative boundary interfaces are considered potential channels of spatial correlation.

The statistical significance of the Moran indices was assessed using a pseudo-significance test based on 999 random permutations. A significance level of α = 0.05 (5%) was adopted to reject the null hypothesis of spatial randomness. No corrections for multiple comparisons, such as Bonferroni or false discovery rate, were applied, since the exploratory nature of this public health surveillance study prioritized sensitivity in detecting risk clusters, avoiding an increase in Type II error (false negatives) that could obscure emerging vulnerable areas.

Thematic maps and the Moran map were created using the publicly available software TerraView 4.2.2, developed by the National Institute for Space Research (INPE), and GeoDa, provided by the University of Chicago^20^. The digital municipal boundary mesh for the Northern region was obtained from IBGE^21^.

As the data were secondary, publicly available, and contained no identifiable subjects, the project was not submitted to the Research Ethics Committee

### Data availability statement

The full dataset supporting the results of this study is available upon request from the corresponding author. The dataset is not publicly available due to the integration of databases carried out by the authors.

## RESULTS

With a resident population of approximately 16.5 million people, 319,228 live births were recorded in 2018 and 289,158 in 2022 across the entire Northern region.

**Figure 2:**
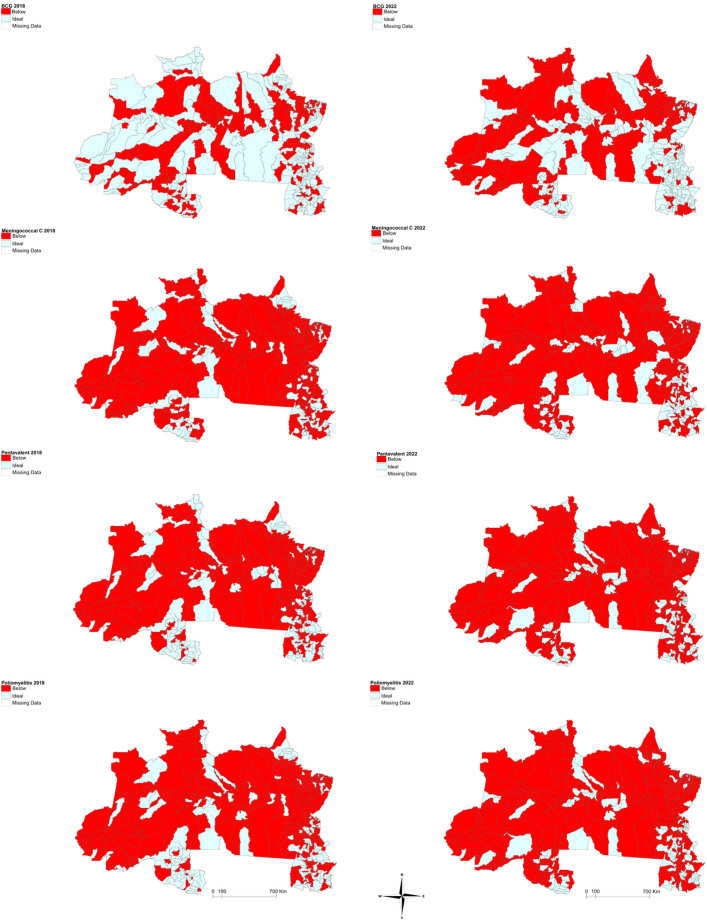
Thematic maps for vaccination coverage. Northern Region, Brazil, 2018 and 2022.

**Figure 3:**
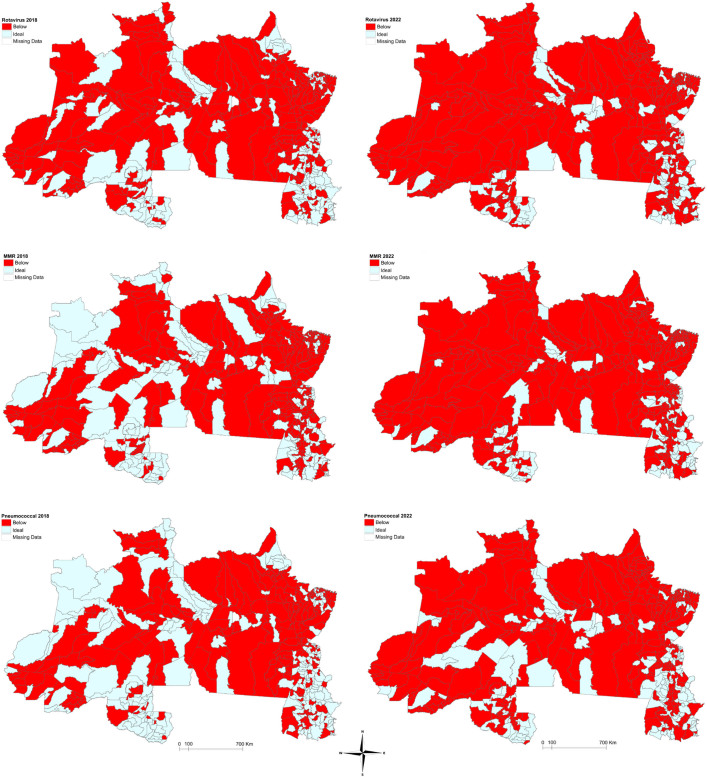
Thematic maps for the immunobiologicals with the largest declines in vaccination coverage. Northern Region, Brazil, 2018 and 2022.

The territory, which comprises 450 municipalities, showed a generalized decline in VC between 2018 and 2022, except for the meningococcal C vaccine (Figure 2). In 2018, 203 municipalities (45.1%) reached the Ministry of Health’s target for rotavirus, a number that fell to 138 (30.7%) in 2022. Pneumococcal coverage decreased from 235 municipalities (52.2%) to 176 (39.1%), and measles-mumps-rubella (MMR) coverage from 190 (42.2%) to 129 (28.7%). BCG coverage fell from 262 municipalities (58.2%), meeting the target in 2018, to 250 (55.6%) in 2022. The pentavalent vaccine followed a similar trajectory, with 174 municipalities (38.7%) in 2018 and 143 (31.8%) in 2022. Poliomyelitis VC decreased from 175 municipalities (38.9%) to 140 (31.1%). Contrasting this predominantly negative trend, the meningococcal C vaccine was the only one to increase, from 133 municipalities (29.6%) in 2018 to 149 (33.1%) in 2022. The largest percentage declines occurred for rotavirus (14.4%), MMR (13.5%), and pneumococcal (13.1%) (Figure 3). Mean, minimum, maximum, and standard deviation values for the study variables are presented in Table 1.

**Table 1:** Minimum, maximum, mean, and standard deviation values of vaccination coverages and of maternal, newborn, and socioeconomic variables. Northern Region, 2018 and 2022.

Variable	Min-Max (%)	Mean (%)	Standard deviation
Poliomyelitis 2018	22.84–223.83	89.58	24.33
Poliomyelitis 2022	19.90–208.69	84.08	26.14
Meningococcal C 2018	26.11–193.15	85.65	24.29
Meningococcal C 2022	18.90–182.60	86.85	24.58
Rotavirus 2018	25.81–388.21	89.67	26.58
Rotavirus 2022	16.91–169.56	81.49	25.18
Pneumococcal 2018	26.70–210.95	97.23	23.40
Pneumococcal 2022	19.23–175.61	90.94	25.54
BCG 2018	7.90–204.97	96.02	29.82
BCG 2022	3.02–311.76	101.82	43.85
Pentavalent 2018	13.90–219.17	87.13	27.43
Pentavalent 2022	18.74–204.34	84.64	25.89
MMR 2018	27.60–288.22	93.18	25.91
MMR 2022	17.33–212.60	84.67	27.30
*Per capita* GDP	3,585,40–118,954,21	12,245,29	9,785,22
Brazilian Deprivation Index	1.13–2.61	0.69	0.69
Schooling 2018	0.00–93.33	49.47	35.38
Schooling 2022	0.00–100.00	54.99	38.46
Proportion of Indigenous People 2018	0.00–95.51	5.42	15.27
Proportion of Indigenous People 2022	0.00–98.17	5.79	15.99
Weight at birth 2018	0.00–18.70	7.17	2.56
Weight at birth 2022	0.00–27.27	8.13	3.01
Term births 2018	0.00–100.00	56.68	38.61
Term births 2022	0.00–97.29	56.87	38.73

In 2022, across the entire stretch of Brazil’s border with French Guiana, Suriname, Guyana, Venezuela, Colombia, and Peru, only two municipalities reached the target coverage for rotavirus and four for poliomyelitis, pentavalent, and MMR. Specifically on the border with Venezuela, no municipality met the target for rotavirus and BCG, while only one reached the target for the other five vaccines. All municipalities on the borders with Suriname and French Guiana were below the target for all immunobiologicals except BCG.

In general, municipalities that met coverage targets in 2022 were concentrated in the states of Tocantins and Rondônia. No state showed improvement in coverage for rotavirus, pneumococcal, poliomyelitis, or MMR between 2018 and 2022.

The bivariate Moran’s I indices were positive and significant (*p* < 0.05) for all variables in this study in 2018 and 2022, indicating spatial autocorrelation and the presence of clusters (Table 2).

**Table 2:** Values of Moran’s I indices — univariate for vaccination coverages and bivariate with *per capita* income and the Brazilian Deprivation Index, with p-values in parentheses. Northern Region, 2018 and 2022.

	Univariate MI	Per capita GDP	IBP
Poliomyelitis 2018	0.132 (0.001)	0.064 (0.005)	−0.238 (0.001)
Poliomyelitis 2022	0.260 (0.001)	0.099 (0.001)	−0.290 (0.001)
Meningococcal C 2018	0.114 (0.001)	0.061 (0.003)	−0.201 (0.001)
Meningococcal C 2022	0.244 (0.001)	0.101 (0.001)	−0.285 (0.001)
Rotavirus 2018	0.053 (0.049)	0.060 (0.010)	−0.212 (0.001)
Rotavirus 2022	0.308 (0.001)	0.108 (0.001)	−0.322 (0.001)
Pneumococcal 2018	0.060 (0.047)	0.048 (0.014)	−0.164 (0.001)
Pneumococcal 2022	0.246 (0.001)	0.089 (0.001)	−0.251 (0.001)
BCG 2018	0.128 (0.001)	0.020 (0.184)	−0.040 (0.040)
BCG 2022	0.314 (0.001)	0.095 (0.001)	−0.232 (0.001)
Pentavalent 2018	0.231 (0.001)	0.078 (0.002)	−0.252 (0.001)
Pentavalent 2022	0.267 (0.001)	0.098 (0.001)	−0.281 (0.001)
MMR 2018	0.058 (0.039)	0.008 (0.324)	−0.092 (0.001)
MMR 2022	0.174 (0.001)	0.096 (0.001)	−0.250 (0.001)

MI: Moran’s I indices; GDP: gross domestic product; IBP: Brazilian Deprivation Index.

All bivariate Moran’s I values between VC and GDP *per capita* were positive and significant, except for BCG and MMR in 2018. This indicates that municipalities with high VC tend to be spatially close to municipalities with high GDP *per capita*, and similarly, municipalities with low VC tend to be near others with low GDP *per capita* (Table 3).

**Table 3:** Values of the bivariate Moran’s I for vaccination coverages and study variables, with p-values in parentheses. Northern Region, 2018 and 2022.

	Schooling	Proportion of Indigenous people	Weight at birth	Term births
Poliomyelitis 2018	0.349 (0.001)	−0.025 (0.127)	−0.028 (0.080)	0.324 (0.001)
Poliomyelitis 2022	0.306 (0.001)	−0.178 (0.001)	0.011 (0.283)	0.260 (0.001)
Meningococcal C 2018	0.283 (0.001)	0.014 (0.244)	−0.038 (0.027)	0.276 (0.001)
Meningococcal C 022	0.267 (0.001)	−0.205 (0.001)	−0.018 (0.232)	0.226 (0.001)
Rotavirus 2018	0.286 (0.001)	−0.012 (0.282)	0.005 (0.367)	0.259 (0.001)
Rotavirus 2022	0.248 (0.001)	−0.255 (0.001)	−0.004 (0.441)	0.190 (0.001)
Pneumococcal 2018	0.317 (0.001)	0.020 (0.177)	0.001 (0.472)	0.302 (0.001)
Pneumococcal 2022	0.292 (0.001)	−0.183 (0.001)	0.003 (0.415)	0.248 (0.001)
BCG 2018	0.210 (0.001)	0.099 (0.001)	0.043 (0.018)	0.201 (0.001)
BCG 2022	0.317 (0.001)	−0.181 (0.001)	−0.013 (0.266)	0.272 (0.001)
Pentavalent 2018	0.400 (0.001)	0.015 (0.241)	−0.004 (0.434)	0.380 (0.001)
Pentavalent 2022	0.316 (0.001)	−0.167 (0.001)	0.009 (0.308)	0.272 (0.001)
MMR 2018	0.258 (0.001)	0.105 (0.001)	−0.031 (0.053)	0.262 (0.001)
MMR 2022	0.299 (0.001)	−0.115 (0.001)	0.007 (0.342)	0.260 (0.001)

The bivariate Moran’s I between VC and the IBP was negative and highly significant for all immunobiologicals, indicating an inverse association in which municipalities with high VC tend to be near others with low IBP. The index value intensified, as with GDP *per capita*, signaling that spatial inequality in coverage associated with economic and deprivation indicators worsened between 2018 and 2022.

Additionally, a significant positive spatial correlation was found between VC and maternal education. Although this relationship weakened for most vaccines, it strengthened for BCG and MMR.

The spatial relationship between VC and the proportion of Indigenous population reversed from 2018 to 2022. While in 2018 the association was mostly non-significant (except for a weak positive correlation for BCG and MMR), in 2022 a strong, significant negative correlation emerged for all vaccines.

Regarding the low birth weight rate, the bivariate spatial correlation analysis did not identify a consistent or statistically significant association with VC in either of the analyzed perios. In contrast, the term birth rate showed a strong, positive, and significant linkage, suggesting clusters in which high coverage coincides with high term-birth rates. The strength of this association decreased for almost all vaccines from 2018 to 2022, with BCG being the only exception.

**Figure 4:**
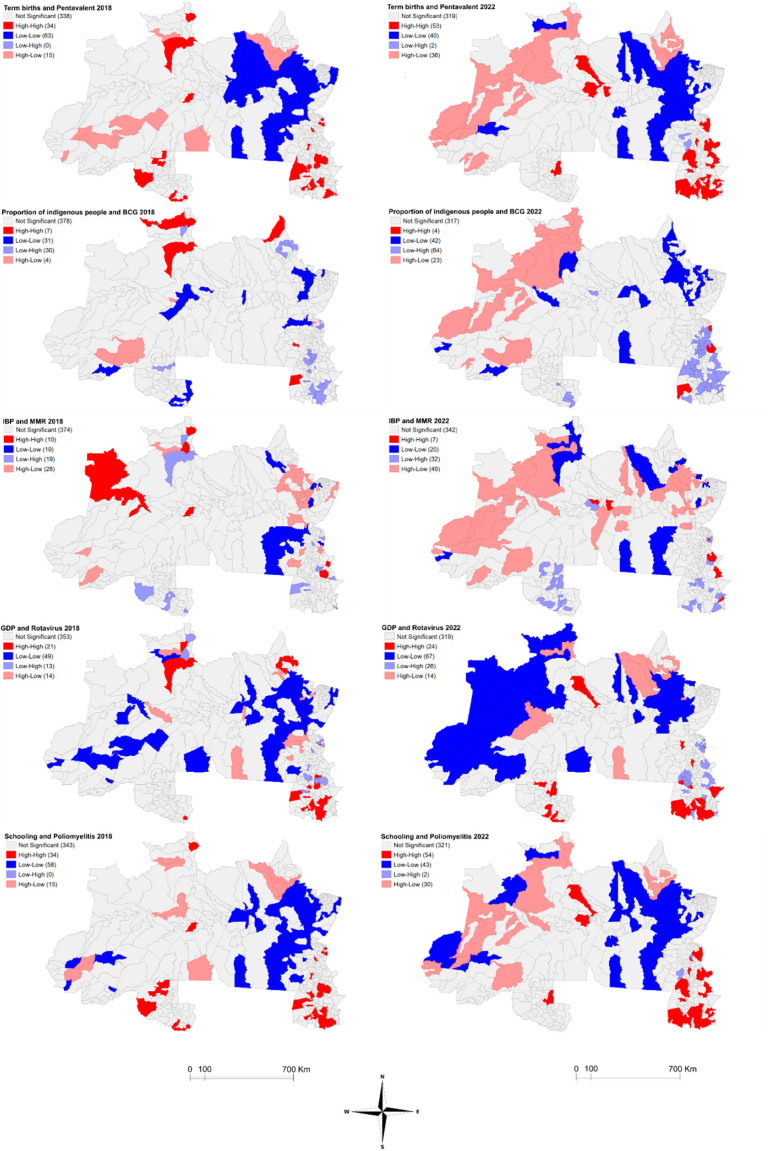
Bivariate local cluster (BiLISA) maps for vaccination coverages and socioeconomic and maternal variables. Northern Region, Brazil, 2018 and 2022. GDP: gross domestic product; IBP: Brazilian Deprivation Index.

The bivariate spatial analysis revealed clear territorial segregation in the Northern region (Figure 4). An extensive low–low cluster was observed in the state of Pará, where low VC coincided with low maternal schooling and low term-birth indicators. The pattern repeated in Amazonas, with a large low–high cluster linking low MMR coverage to a high IBP in 2022, and low rotavirus coverage to lower GDP *per capita*. In Roraima and in western Amazonas, low VC coincided with a higher proportion of Indigenous population in the same year. In contrast, a mutual-benefit (high–high) scenario emerged in Tocantins, where high-VC clusters consistently aligned with better socioeconomic indicators, such as higher GDP *per capita*, maternal schooling, and term-birth rates. It should be noted that the observed associations are at the aggregated municipal level and do not permit individual-level causal inference.

## DISCUSSION

To our knowledge, this is the first study to analyze the spatial distribution of VC in the Northern region, using geoprocessing to correlate the set of immunobiologicals with socioeconomic and maternal indicators in the pre- and post-pandemic periods (2018 and 2022).

This study reveals a widespread decline in vaccination coverage, consistent with recent studies. According to a 2025 survey released by the United Nations Children’s Fund (UNICEF) and the World Health Organization (WHO), Brazil is among the 20 countries with the highest number of unvaccinated children^22^. Recurrent failures in the supply of immunobiologicals and the COVID-19 pandemic are repeatedly cited in the survey as factors that contributed to the reduction in childhood vaccination.

Although the COVID-19 pandemic imposed global disruptions on immunization programs, it is imperative to avoid homogenizing this temporal block. Instead, we propose stratification into two distinct intervals, each characterized by idiosyncratic causal etiologies for the decline in vaccination indicators. The first period, corresponding to 2020 and 2021, was predominantly characterized by physical and operational barriers: social isolation and distancing measures restricted access to health facilities, fear of infection at health services reduced demand for vaccination, and the massive redeployment of teams and resources to combat COVID-19 compromised the provision of routine services^23,24,25^. In contrast, the 2022 scenario reflected a crisis of confidence and governance. Vaccine hesitancy intensified during this period, fueled by structured misinformation and the strengthening of the anti-vaccine movement—phenomena amplified by the politicization of health^26,27^. Finally, the health system faced a backlog of demand, with millions of children accumulating delays in vaccination schedules that were not adequately recovered even after restrictions were relaxed^28^.

Low VC in border municipalities, combined with intense migratory flows, creates an epidemiological scenario conducive to the reemergence of vaccine-preventable diseases. Studies on viral hepatitis in immigrants and refugees show high prevalences of exposure to hepatitis A and B viruses, with substantial vaccination gaps in children^11,12^. Another example of this risk was the reintroduction of measles in Brazil, directly linked to Venezuelan migration in the context of low MMR coverage in recipient municipalities in the North^9^. The movement of susceptible populations through areas with barriers to healthcare access, such as border regions, acts as a corridor for pathogen dissemination.

The bivariate analysis with socioeconomic indicators indicates that the spatial distribution of childhood vaccination in the Northern region reflects deep social inequities. For all vaccines, the bivariate Moran’s I values between VC and GDP *per capita* and between VC and the IBP increased from 2018 to 2022, indicating that the spatial concentration of high VC in wealthier areas and low coverage in poorer areas became more pronounced in 2022. This finding suggests that the pandemic’s impact was asymmetric, disproportionately affecting the most vulnerable municipalities and deepening inequalities in access to immunization.

Nonetheless, the presence of outlier municipalities (characterized by high social vulnerability, yet with elevated VC) represents a phenomenon of “health resilience” that warrants analytical attention. These municipalities show that socioeconomic inequality is not an insurmountable determinant of health outcomes. This scenario is consistent with the literature, which describes the strength of Primary Health Care and the widespread work of Community Health Workers as a key factor in vaccination success in poor territories^29,30,31^. The extensive reach of the Unified Health System, through the Family Health Strategy and the active outreach of Community Health Workers, mitigates socioeconomic inequities, protecting vulnerable populations even in adverse contexts.

One of the most alarming findings of this study was the reversal in the correlation between VC and the proportion of Indigenous population. In 2018 there was no significant spatial association; however, by 2022 this correlation became strongly negative and statistically significant for all immunobiologicals analyzed. This shift reveals the emergence of a new spatial dynamic, with vulnerability clusters linking low coverage to areas with higher Indigenous presence. The decline in VC among Indigenous populations in Brazil is multifactorial, involving access barriers, socioeconomic inequalities, management failures, misinformation, and political instability that undermine vaccine confidence. Geographic and operational barriers are particularly relevant, as the social and geographic isolation of Indigenous communities hinders the implementation of vaccination strategies and limits regular access to health services, especially in remote and hard-to-reach areas^32,33^. The absence of health professional visits is also associated with higher rates of incomplete vaccination schedules, indicating that the active presence of health teams is critical for vaccination adherence^33^.

Moreover, the observed reversal reflects a paradigm shift in Indigenous health management, characterized by the progressive dismantling of the Special Secretariat for Indigenous Health (SESAI)^34^ and the weakening of territorial protection policies^35,36^. The expansion of illegal mining on Indigenous lands, which increased by 129% between 2013 and 2021 and intensified especially after 2018, created an environment of violence and insecurity that made it impossible for health professionals to enter critical areas. As evidenced by the Yanomami humanitarian crisis, this process consolidated vulnerability clusters in which geographic isolation was exacerbated by the absence of state protection^37,38,39^.

The positive spatial correlation between maternal schooling and VC is consistent, at the aggregate level, with literature that identifies education as a determinant of health literacy and proactive care-seeking behaviors. Although ecological analysis does not allow inference about individual behavior, the co-occurrence of high maternal schooling rates and high VC in the same territories supports studies that identify maternal education as a robust factor for better child health outcomes^40,41,42,43,44,45,46^. A global meta-analysis showed that the odds of complete vaccination are more than twice as high among children of mothers with secondary or higher education, although the effect varies by socioeconomic and geographic context^47^. The attenuation of this association may be attributed to a systemic decline in VC that affected all social strata, reducing the differential between educational groups.

The spatial patterns identified have direct implications for health surveillance, and low-coverage areas represent an imminent risk for the resurgence of vaccine-preventable diseases that have already been eliminated in the country. Municipal-level analysis, as conducted in this study, is therefore a strategic tool for planning public health actions, allowing managers to target resources and intensified vaccination campaigns to higher-risk territories.

The clusters are not random; they are the spatial manifestation of deep and persistent socioeconomic and structural inequalities that characterize the Northern region. Historically, the region faces a fragile health system, poor infrastructure, and access barriers, especially for its vast and often isolated populations—such as Indigenous, riverside (*ribeirinha*), and quilombola communities. The COVID-19 pandemic did not create these disparities but deepened and starkly exposed them, producing an epidemiological risk scenario that requires urgent, territorially focused, and culturally sensitive actions.

This study may have limitations inherent to its ecological design, which precludes causal inference at the individual level. In addition, the use of secondary DATASUS data is subject to possible biases and inconsistencies in the recording of information, such as artificially inflated (over 100%) or underestimated coverage rates. VCs above 100% may be related to the numerator being based on the place of vaccine administration rather than the vaccinee’s place of residence, as well as to outdated population estimates in the denominator. In this study, we chose to keep the original recorded values without truncation in order to preserve the statistical variability required for sensitive cluster detection and to avoid the bias of homogenization, or “ceiling effect,” that would occur by equating municipalities with distinct performances. This approach also allows reflecting the actual volume of doses administered and capturing genuine spatial phenomena, such as service polarization in neighboring municipalities and border effects.

However, the robustness of the spatial analysis and the statistical significance of the patterns observed lend validity to our findings, reinforce the urgency of the problem, and provide evidence to inform the planning and strategic targeting of public health actions toward higher-risk areaso.
