# Supplementary material for: Childhood vaccination coverage and socioeconomic inequalities in the Northern region of Brazil
Source: Rev Bras Epidemiol. 2026 Jul 27;29:e260027. doi: 10.1590/1980-549720260027 (PMC13405557; doi:10.1590/1980-549720260027)
Supplement: Translation

# Translation: Cobertura vacinal infantil e desigualdades socioeconômicas na região Norte do Brasil

**DOI:** 10.1590/1980-549720260027.2

**Authors:** Eduarda de Paulo Costa, Luiz Fernando Costa Nascimento

**Affiliations:** I Universidade de São Paulo, Faculdade de Medicina – São Paulo (SP), Brasil.; II Universidade Estadual Paulista Júlio de Mesquita Filho, Faculdade de Engenharia e Ciências – Guaratinguetá (SP), Brasil.

**Keywords:** Cobertura vacinal, Análise espacial, Desigualdades em saúde, Fatores socioeconômicos, Saúde da criança

## Abstract

**Objetivo:**

Avaliar a variação espaço-temporal da cobertura vacinal de imunobiológicos infantis nos municípios da região Norte do Brasil, comparando os cortes transversais de 2018 e 2022, bem como sua associação com variáveis socioeconômicas e maternas.

**Métodos:**

Trata-se de um estudo ecológico comparativo com dados de cobertura para sete imunobiológicos nos municípios da região Norte nos anos de 2018 e 2022. Dados foram obtidos do Departamento de Informática do Sistema Único de Saúde. Utilizou-se análise espacial (Índice de Moran global, bivariado e local) para verificar a associação entre coberturas vacinais e variáveis independentes (PIB *per capita*, Índice Brasileiro de Privação, escolaridade materna, proporção de indígenas, nascidos a termo e peso ao nascer).

**Resultados:**

Houve um declínio generalizado na cobertura vacinal ao comparar os dois períodos, com exceção da vacina meningocócica C. A vacinação associou-se positivamente ao PIB *per capita* e à escolaridade materna e negativamente ao Índice de Privação, com intensificação dessas associações no período. A correlação espacial entre imunização e proporção de população indígena, antes não significativa, tornou-se fortemente negativa em 2022. Identificaram-se agrupamentos (*clusters*) de baixa imunização e vulnerabilidade socioeconômica majoritariamente nos estados do Pará e Amazonas, e de alta imunização e melhores indicadores em Tocantins e Rondônia.

**Conclusão:**

A queda da vacinação infantil na região Norte é um fenômeno espacialmente estruturado que reflete e aprofunda desigualdades socioeconômicas. A formação de *clusters* de vulnerabilidade, especialmente em áreas próximas à fronteira brasileira, representa um risco para a reemergência de doenças imunopreveníveis e exige políticas públicas de saúde territorialmente focadas.

## INTRODUÇÃO

A vacinação é uma das estratégias de saúde pública mais eficazes e custo-efetivas na redução da mortalidade infantil, na prevenção de doenças e na promoção do desenvolvimento saudável na infância. Desde a implementação de estratégias de vacinação em larga escala, como o Programa Ampliado de Imunização da Organização Mundial da Saúde (OMS), estima-se que mais de 154 milhões de mortes foram evitadas em todo o mundo, sendo 146 milhões em crianças menores de 5 anos e 101 milhões em lactentes^1–3^.

No Brasil, o Programa Nacional de Imunizações, instituído pelo Ministério da Saúde em 1973, oferece gratuitamente um amplo calendário vacinal, sendo reconhecido internacionalmente por sua capacidade de erradicação de doenças imunopreveníveis, como a erradicação da poliomielite selvagem e da circulação do vírus da rubéola, além da significativa redução nos casos e óbitos por doenças como difteria, tétano, sarampo e hepatite B^4^.

Entretanto, desde 2016, observa-se uma redução na cobertura vacinal (CV) em todo o país^4^. Essa tendência se acentuou durante a pandemia de COVID-19, que sobrecarregou o sistema de saúde, restringiu a mobilidade e gerou receio de exposição ao vírus nos serviços de saúde, resultando em quedas abruptas nas taxas de vacinação^5^. A heterogeneidade regional é marcante, com as regiões Norte e Nordeste apresentando os maiores declínios^6^, embora o fenômeno seja nacional e afete inclusive áreas historicamente com altas coberturas^7^.

A imigração proveniente de países vizinhos, especialmente da Venezuela e da Guiana, tem impactado significativamente a saúde pública e a CV nos municípios da fronteira Norte brasileira. O aumento da mobilidade humana, motivado por crises humanitárias e atividades econômicas como a mineração, está associado à elevação de casos importados de doenças transmissíveis, como malária, hepatites virais e sarampo, além de desafios na manutenção de altas CVs^8–12^.

O cálculo da CV em níveis estaduais ou nacionais pode ocultar áreas de vulnerabilidade em decorrência da diluição de heterogeneidades espaciais e sociais por médias agregadas. Sem uma análise granular, intervenções podem ser mal direcionadas, perpetuando desigualdades e vulnerabilidades^13–17^. Uma alternativa metodológica é a análise espacial em escala municipal, que permite mapear áreas de risco epidemiológico e facilita a detecção de determinantes sociais subjacentes.

Nesse contexto, este estudo tem como objetivo avaliar a variação espacial e temporal da CV de imunobiológicos administrados na infância nos municípios da região Norte do Brasil entre os anos de 2018 e 2022, bem como sua associação com variáveis socioeconômicas e maternas.

## MÉTODOS

Trata-se de um estudo ecológico com dados da CV em 450 municípios nos sete estados da região Norte (Figura 1)^18^ nos anos de 2018 e 2022, obtidos no Departamento de Informática do Sistema Único de Saúde (DATASUS)^19^.

**Figura 1:** Mapa político do Brasil, identificando a Região Norte e seus respectivos estados: Acre (AC), Amapá (AP), Amazonas (AM), Pará (PA), Rondônia (RO), Roraima (RR) e Tocantins (TO).

A seleção de imunobiológicos compreendeu aqueles preconizados para a vacinação infantil, sendo eles: BCG (tuberculose), pentavalente (difteria, tétano, coqueluche, hepatite B e *Haemophilus influenzae* tipo b), poliomielite, pneumocócica, rotavírus humano, meningocócica C e tríplice viral (sarampo, caxumba e rubéola).

As CVs (variável dependente) foram obtidas em porcentagem por antígeno em cada município. Os valores médios foram comparados entre os anos de 2018 e 2022. Posteriormente, identificaram-se as localidades que não atingiram as coberturas recomendadas pelo Ministério da Saúde: 90% para a BCG e o rotavírus e 95% para os demais imunobiológicos.

Os indicadores socioeconômicos (variáveis independentes) escolhidos foram produto interno bruto (PIB) *per capita* e Índice Brasileiro de Privação (IBP), além de variáveis maternas como instrução (oito ou mais anos de escolaridade), raça (proporção de indígenas entre os nascidos vivos), baixo peso ao nascer da criança (<2500 g) e duração da gestação (nascimentos a termo), todas em porcentagem.

Criado por pesquisadores da Fiocruz Bahia e da Universidade de Glasgow, o IBP mensura a privação material com base em informações sobre renda, escolaridade e condições de moradia no nível da menor unidade territorial utilizada pelo Instituto Brasileiro de Geografia e Estatística (IBGE), os setores censitários, o que permite identificar bolsões de pobreza dentro dos próprios municípios. Esse índice pode assumir valores positivos e negativos, de modo que um valor mais positivo indica maior privação.

Para a análise da dinâmica espacial, foram estimados os índices de Moran global e respectivos p-valores, visando avaliar a autocorrelação espacial entre os municípios segundo determinada variável. Também foram calculados os Índices de Moran bivariado e local (LISA).

O Índice de Moran cria uma medida da associação espacial entre um agrupamento de dados numa escala que varia de −1 a +1. Valores positivos (autocorrelação positiva) indicam similaridade entre os valores de uma característica e sua localização espacial; valores negativos (autocorrelação negativa) indicam dissimilaridade; e valores próximos a zero indicam que a distribuição no espaço é aleatória. O Índice de Moran global mede a autocorrelação espacial considerando todo o conjunto de dados, ou seja, avalia se existe um padrão espacial global em toda a área de estudo. O Índice de Moran bivariado é utilizado para avaliar a autocorrelação espacial entre duas variáveis distintas, verificando se valores altos (ou baixos) de uma variável em uma localidade estão associados a valores altos (ou baixos) de outra variável nas localidades vizinhas. O LISA, por sua vez, indica se aquela localidade e seus vizinhos apresentam valores similares (*clusters* do tipo alto-alto ou baixo-baixo) ou discrepantes (alto-baixo ou baixo-alto), possibilitando a identificação de áreas com padrões espaciais distintos dentro do conjunto global.

Para a definição da vizinhança espacial, utilizou-se uma matriz de pesos por contiguidade do tipo *Queen* de primeira ordem, padronizada por linha, considerando como vizinhos os municípios que compartilham fronteiras ou vértices comuns. Essa escolha justifica-se pela extrema heterogeneidade das áreas territoriais dos municípios da Região Norte, o que inviabiliza o uso de matrizes baseadas em distância fixa entre centroides, as quais poderiam gerar falsos isolamentos em municípios extensos ou conexões excessivas em áreas mais densas. A matriz Queen foi selecionada por ampliar a conectividade topológica entre as unidades espaciais, reduzindo o impacto das geometrias irregulares da malha municipal analisada e assegurando que todas as interfaces de fronteira administrativa fossem consideradas como potenciais canais de correlação espacial.

A significância estatística dos índices de Moran foi aferida mediante o teste de pseudo-significância baseado em 999 permutações aleatórias. Adotou-se um nível de significância de α=0,05 (5%) para a rejeição da hipótese nula de aleatoriedade espacial. Não foram aplicadas correções para múltiplas comparações, como Bonferroni ou taxa de descobertas falsas (*false discovery rate*), visto que o caráter exploratório do estudo em vigilância em saúde prioriza a sensibilidade na detecção de aglomerados de risco, evitando o aumento do erro do tipo II (falsos negativos) que poderia mascarar áreas vulneráveis em formação.

Os mapas temáticos e o mapa de Moran foram construídos utilizando os *softwares* de acesso público TerraView 4.2.2, desenvolvido pelo Instituto Nacional de Pesquisas Espaciais (INPE), e GeoDa, disponibilizado pela Universidade de Chicago^20^. A malha digital dos municípios da região Norte foi obtida pelo IBGE^21^.

Por serem dados secundários, de acesso público e com sujeitos não identificáveis, o projeto não foi submetido ao Comitê de Ética em Pesquisa.

### Declaração de disponibilidade de dados

Todo o conjunto de dados que dá suporte aos resultados deste estudo está disponível mediante solicitação ao autor correspondente. O conjunto de dados não está publicamente disponível devido à integração de bancos de dados realizados pelos autores.

## RESULTADOS

Com uma população residente de aproximadamente 16,5 milhões de pessoas, registraram-se 319.228 nascidos vivos em 2018 e 289.158 em 2022 em toda a região Norte.

O território, que possui 450 municípios, apresentou um declínio generalizado na CV entre 2018 e 2022, à exceção da vacina meningocócica C (Figura 2). Em 2018, 203 municípios (45,1%) alcançaram a meta preconizada pelo Ministério da Saúde para rotavírus, número que caiu para 138 (30,7%) em 2022. A cobertura da pneumocócica diminuiu de 235 municípios (52,2%) para 176 (39,1%), e a tríplice viral de 190 (42,2%) para 129 (28,7%). A vacina BCG teve redução de 262 municípios (58,2%) que atingiram a meta em 2018 para 250 (55,6%) em 2022. A pentavalente seguiu trajetória semelhante, com 174 municípios (38,7%) em 2018 e 143 (31,8%) em 2022. A vacina contra poliomielite teve o número de municípios reduzido de 175 (38,9%) para 140 (31,1%). Contrastando essa tendência predominantemente negativa, a vacina meningocócica C foi a única que apresentou aumento, passando de 133 municípios (29,6%) em 2018 para 149 (33,1%) em 2022. As maiores quedas percentuais ocorreram para rotavírus (14,4%), tríplice viral (13,5%) e pneumocócica (13,1%) (Figura 3). Os valores médios, mínimos, máximos e o desvio padrão das variáveis deste estudo são apresentados na Tabela 1.

**Figura 2:** Mapas temáticos para a cobertura vacinal. Região Norte, Brasil, 2018 e 2022. Missing data: dados ausentes.

**Figura 3:** Mapas temáticos para os imunobiológicos com as maiores quedas em cobertura vacinal. Região Norte, Brasil, 2018 e 2022. Missing data: dados ausentes.

**Tabela 1:** Valores mínimo, máximo, média e desvio padrão das coberturas vacinais e das variáveis materna, do recém-nascido e socioeconômicas. Região Norte, 2018 e 2022.

Variável	Min-Max (%)	Média (%)	Desvio padrão
Poliomielite 2018	22,84–223,83	89,58	24,33
Poliomielite 2022	19,90–208,69	84,08	26,14
Meningocócica C 2018	26,11–193,15	85,65	24,29
Meningocócica C 2022	18,90–182,60	86,85	24,58
Rotavírus 2018	25,81–388,21	89,67	26,58
Rotavírus 2022	16,91–169,56	81,49	25,18
Pneumocócica 2018	26,70–210,95	97,23	23,40
Pneumocócica 2022	19,23–175,61	90,94	25,54
BCG 2018	7,90–204,97	96,02	29,82
BCG 2022	3,02–311,76	101,82	43,85
Pentavalente 2018	13,90–219,17	87,13	27,43
Pentavalente 2022	18,74–204,34	84,64	25,89
Tríplice viral 2018	27,60–288,22	93,18	25,91
Tríplice viral 2022	17,33–212,60	84,67	27,30
PIB *per capita*	3,585,40–118,954,21	12,245,29	9,785,22
Índice brasileiro de privação	1,13–2,61	0,69	0,69
Escolaridade 2018	0,00–93,33	49,47	35,38
Escolaridade 2022	0,00–100,00	54,99	38,46
Proporção de indígenas 2018	0,00–95,51	5,42	15,27
Proporção de indígenas 2022	0,00–98,17	5,79	15,99
Peso ao nascer 2018	0,00–18,70	7,17	2,56
Peso ao nascer 2022	0,00–27,27	8,13	3,01
Nascimentos a termo 2018	0,00–100,00	56,68	38,61
Nascimentos a termo 2022	0,00–97,29	56,87	38,73

Em 2022, considerando toda a faixa da fronteira brasileira com Guiana Francesa, Suriname, Guiana, Venezuela, Colômbia e Peru, apenas dois municípios atingiram a CV ideal para rotavírus e quatro para poliomielite, pentavalente e tríplice viral. Especificamente na fronteira com a Venezuela, nenhum município atingiu a meta para rotavírus e BCG, enquanto apenas um alcançou a CV ideal para as outras cinco vacinas. Todos os municípios da fronteira com Suriname e Guiana Francesa estavam abaixo da meta para todos os imunobiológicos, à exceção da BCG.

Em geral, os municípios que atingiram as metas de cobertura em 2022 concentraram-se nos estados de Tocantins e Rondônia. Nenhum estado apresentou melhora na cobertura contra rotavírus, pneumocócica, poliomielite e tríplice viral entre 2018 e 2022.

Os índices de Moran bivariado foram positivos e significativos (p<0,05) para todas as variáveis deste estudo em 2018 e 2022, indicando autocorrelação espacial e presença de *clusters* (Tabela 2).

**Tabela 2:** Valores dos Índices de Moran, univariado das coberturas vacinais e bivariado com as variáveis renda *per capita* e índice brasileiro de privação com p-valor em parênteses. Região Norte, 2018 e 2022.

	IM univariado	PIB per capita	IBP
Poliomielite 2018	0,132 (0,001)	0,064 (0,005)	−0,238 (0,001)
Poliomielite 2022	0,260 (0,001)	0,099 (0,001)	−0,290 (0,001)
Meningocócica C 2018	0,114 (0,001)	0,061 (0,003)	−0,201 (0,001)
Meningocócica C 2022	0,244 (0,001)	0,101 (0,001)	−0,285 (0,001)
Rotavírus 2018	0,053 (0,049)	0,060 (0,010)	−0,212 (0,001)
Rotavírus 2022	0,308 (0,001)	0,108 (0,001)	−0,322 (0,001)
Pneumocócica 2018	0,060 (0,047)	0,048 (0,014)	−0,164 (0,001)
Pneumocócica 2022	0,246 (0,001)	0,089 (0,001)	−0,251 (0,001)
BCG 2018	0,128 (0,001)	0,020 (0,184)	−0,040 (0,040)
BCG 2022	0,314 (0,001)	0,095 (0,001)	−0,232 (0,001)
Pentavalente 2018	0,231 (0,001)	0,078 (0,002)	−0,252 (0,001)
Pentavalente 2022	0,267 (0,001)	0,098 (0,001)	−0,281 (0,001)
Tríplice viral 2018	0,058 (0,039)	0,008 (0,324)	−0,092 (0,001)
Tríplice viral 2022	0,174 (0,001)	0,096 (0,001)	−0,250 (0,001)

IM: Índice de Moran; PIB: produto interno bruto; IBP: Índice brasileiro de privação.

Todos os valores do Índice de Moran bivariado entre CV e PIB *per capita* foram positivos e significativos, com exceção da BCG e tríplice viral em 2018. Isso indica que municípios com alta CV tendem a estar espacialmente próximos de municípios com alto PIB *per capita*. Analogamente, municípios com baixa CV tendem a estar próximos a outros com baixo PIB *per capita* (Tabela 3).

**Tabela 3:** Valores do Índice de Moran bivariado das coberturas vacinais e variáveis do estudo com p-valor em parênteses. Região Norte, 2018 e 2022.

	Escolaridade	Proporção de indígenas	Peso ao nascer	Nascimentos a termo
Poliomielite 2018	0,349 (0,001)	−0,025 (0,127)	−0,028 (0,080)	0,324 (0,001)
Poliomielite 2022	0,306 (0,001)	−0,178 (0,001)	0,011 (0,283)	0,260 (0,001)
Meningocócica C 2018	0,283 (0,001)	0,014 (0,244)	−0,038 (0,027)	0,276 (0,001)
Meningocócica C 022	0,267 (0,001)	−0,205 (0,001)	−0,018 (0,232)	0,226 (0,001)
Rotavírus 2018	0,286 (0,001)	−0,012 (0,282)	0,005 (0,367)	0,259 (0,001)
Rotavírus 2022	0,248 (0,001)	−0,255 (0,001)	−0,004 (0,441)	0,190 (0,001)
Pneumocócica 2018	0,317 (0,001)	0,020 (0,177)	0,001 (0,472)	0,302 (0,001)
Pneumocócica 2022	0,292 (0,001)	−0,183 (0,001)	0,003 (0,415)	0,248 (0,001)
BCG 2018	0,210 (0,001)	0,099 (0,001)	0,043 (0,018)	0,201 (0,001)
BCG 2022	0,317 (0,001)	−0,181 (0,001)	−0,013 (0,266)	0,272 (0,001)
Pentavalente 2018	0,400 (0,001)	0,015 (0,241)	−0,004 (0,434)	0,380 (0,001)
Pentavalente 2022	0,316 (0,001)	−0,167 (0,001)	0,009 (0,308)	0,272 (0,001)
Tríplice viral 2018	0,258 (0,001)	0,105 (0,001)	−0,031 (0,053)	0,262 (0,001)
Tríplice viral 2022	0,299 (0,001)	−0,115 (0,001)	0,007 (0,342)	0,260 (0,001)

O Índice de Moran bivariado entre CV e IBP foi negativo e altamente significativo para todos os imunobiológicos, indicando uma associação inversa, em que municípios com alta CV tendem a estar próximos de outros com baixo IBP. O valor do índice intensificou-se, tal como para o PIB *per capita*, sinalizando que a desigualdade espacial na cobertura, associada a indicadores econômicos e de privação, acentuou-se entre 2018 e 2022.

Adicionalmente, identificou-se correlação espacial positiva significativa entre CV e escolaridade materna. Embora essa relação tenha se atenuado para a maioria das vacinas, observou-se fortalecimento para BCG e tríplice viral.

A relação espacial entre CV e proporção de população indígena inverteu-se de 2018 para 2022. Enquanto em 2018 a associação era majoritariamente não significante (exceto por uma fraca correlação positiva para BCG e tríplice viral), em 2022 emergiu um padrão de correlação negativa, forte e significante para todas as vacinas.

Em relação à taxa de baixo peso ao nascer, a análise de correlação espacial bivariada não identificou associação consistente ou estatisticamente relevante com a CV em nenhum dos períodos analisados. Contrariamente, a taxa de nascidos a termo apresentou forte, positiva e significante vinculação, sugerindo *clusters* em que a alta cobertura coincide com altas taxas de nascidos a termo. A força dessa associação diminuiu para quase todas as vacinas de 2018 para 2022, sendo a BCG a única exceção.

A análise espacial bivariada revelou uma nítida segregação territorial na região Norte (Figura 4). Um extenso *cluster* do tipo baixo-baixo foi observado no estado do Pará, onde baixas CVs coincidiram com baixos indicadores de escolaridade e de nascimentos a termo. O padrão repetiu-se no Amazonas, com um grande *cluster* baixo-alto conectando a baixa cobertura da tríplice viral a um alto IBP em 2022, e a baixa cobertura para rotavírus a um menor PIB *per capita*. Em Roraima e no Oeste do Amazonas, a baixa CV coincidiu com uma maior proporção de população indígena no mesmo ano. Em forte contraste, um cenário de vantagem mútua (alto-alto) emergiu no Tocantins, onde *clusters* de alta CV se alinharam consistentemente a melhores indicadores socioeconômicos, como maior PIB *per capita*, escolaridade e taxa de nascimentos a termo. Ressalta-se que as associações observadas ocorrem em nível municipal agregado, não permitindo inferência causal individual.

**Figura 4:** Mapas de *cluster* local bivariado (BiLISA) para as coberturas vacinais e variáveis socioeconômicas e maternas. Região Norte, Brasil, 2018 e 2022. PIB: produto interno bruto; IBP: Índice brasileiro de privação.

## DISCUSSÃO

A nosso conhecimento, este é o primeiro estudo a analisar a distribuição espacial da CV na região Norte, utilizando geoprocessamento para correlacionar o conjunto de imunobiológicos a indicadores socioeconômicos e maternos, no contexto temporal pré e pós-pandemia (2018 e 2022).

O presente trabalho revela um declínio generalizado na CV, de acordo com estudos mais recentes. Segundo levantamento divulgado em 2025 pelo Fundo das Nações Unidas para a Infância (UNICEF) e pela OMS, o Brasil é um dos 20 países com maior número de crianças não vacinadas^22^. Falhas recorrentes no fornecimento de imunobiológicos e a pandemia de COVID-19 são citadas repetidamente no levantamento como fatores que contribuíram para a redução da vacinação infantil.

Ainda que a pandemia de COVID-19 tenha imposto disrupções globais aos programas de imunização, é imperativo evitar a homogeneização deste bloco temporal. Propõe-se, em contrapartida, a estratificação em dois intervalos distintos, caracterizados por etiologias causais idiossincráticas para a queda nos indicadores vacinais. O primeiro momento, correspondente aos anos de 2020 e 2021, foi caracterizado predominantemente por barreiras físicas e operacionais: o isolamento social e as medidas de distanciamento restringiram o acesso às unidades de saúde, o medo de infecção nos serviços de saúde reduziu a procura por vacinação, e o redirecionamento massivo de equipes e recursos para o combate à COVID-19 comprometeu a oferta de serviços de rotina^23–25^. Em contraste, o cenário de 2022 configurou uma crise de confiança e gestão. A hesitação vacinal intensificou-se nesse período, alimentada por desinformação estruturada e pelo fortalecimento do movimento antivacina, fenômenos amplificados pela politização da saúde^26,27^. Finalmente, o sistema de saúde enfrentou o desafio do represamento de demandas (*backlog*), com milhões de crianças acumulando atrasos nos esquemas vacinais que não foram adequadamente recuperados mesmo após a flexibilização das medidas restritivas^28^.

A baixa CV em municípios fronteiriços, somada ao intenso fluxo migratório, cria um cenário epidemiológico propício para a reemergência de doenças imunopreveníveis. Estudos sobre hepatites virais em imigrantes e refugiados mostram prevalências elevadas de exposição aos vírus das hepatites A e B, com falhas vacinais importantes em crianças^11,12^. Outro exemplo desse risco foi a reintrodução do sarampo no Brasil, diretamente ligada à migração venezuelana em um contexto de baixa cobertura da tríplice viral nos municípios receptores do Norte^9^. A circulação de populações suscetíveis em áreas com barreiras de acesso à saúde, como as fronteiras, funciona como um corredor para a disseminação de patógenos.

O resultado da análise bivariada com os indicadores socioeconômicos sinaliza que a distribuição espacial da vacinação infantil na região Norte reflete profundas iniquidades sociais. Para todas as vacinas, o valor do Índice de Moran bivariado da CV com PIB *per capita* e IBP aumentou de 2018 para 2022, indicando que a concentração espacial de altas coberturas vacinais em áreas de maior poder aquisitivo e de baixas coberturas em áreas de menor poder aquisitivo tornou-se mais intensa em 2022. Tal achado sugere que o impacto da pandemia foi assimétrico, afetando desproporcionalmente os municípios mais vulneráveis e aprofundando as desigualdades no acesso à imunização.

Não obstante, a existência de municípios *outliers* (com valores atípicos) com alta vulnerabilidade social e elevadas coberturas vacinais representa um fenômeno de “resiliência em saúde” que merece destaque analítico. Esses municípios demonstram que a desigualdade socioeconômica não é um determinante insuperável dos resultados em saúde. Tal cenário é consistente com a literatura, que descreve a força da Atenção Primária à Saúde e a atuação capilarizada dos Agentes Comunitários de Saúde como fator de sucesso vacinal de territórios pobres^29–31^. A capilaridade do Sistema Único de Saúde, por meio da Estratégia Saúde da Família e da busca ativa pelos Agentes Comunitários de Saúde, mitiga iniquidades socioeconômicas, protegendo populações vulneráveis mesmo em contextos adversos.

Um dos achados mais alarmantes deste estudo foi a inversão na correlação entre a CV e a proporção de população indígena. Em 2018, não havia uma associação espacial significativa; contudo, em 2022, essa correlação tornou-se fortemente negativa e estatisticamente significante para todos os imunobiológicos analisados. Tal evento evidencia o surgimento de uma nova dinâmica espacial, com *clusters* de vulnerabilidade que associam baixa cobertura a áreas com maior presença indígena. O declínio da CV entre indígenas no Brasil é multifatorial, envolvendo barreiras de acesso, desigualdades socioeconômicas, falhas de gestão, desinformação e instabilidade política que afeta a confiança nas vacinas. Barreiras geográficas e operacionais são particularmente relevantes, à medida que o isolamento social e geográfico das comunidades indígenas dificulta a implementação de estratégias de vacinação e limita o acesso regular aos serviços de saúde, especialmente em áreas remotas e de difícil locomoção^32,33^. A ausência de visitas de profissionais de saúde também está associada a maiores taxas de esquemas vacinais incompletos, indicando que a presença ativa de equipes de saúde é determinante para a adesão à vacinação^33^.

Ademais, a inversão observada reflete uma mudança de paradigma na gestão da saúde indígena, caracterizada pela desestruturação progressiva da Secretaria Especial de Saúde Indígena (SESAI)^34^ e pelo enfraquecimento das políticas de proteção territorial^35,36^. A expansão do garimpo ilegal nas terras indígenas, que aumentou 129% entre 2013 e 2021 e se intensificou particularmente após 2018, criou um ambiente de violência e insegurança que inviabilizou a entrada de profissionais de saúde em áreas críticas. Como evidenciado pela crise humanitária Yanomami, esse processo consolidou *clusters* de vulnerabilidade, nos quais o isolamento geográfico foi agravado pela ausência de proteção estatal^37–39^.

A correlação espacial positiva entre o grau de instrução materna e a CV é consistente, em nível agregado, com a literatura que descreve a educação como determinante de letramento em saúde e de comportamentos proativos na busca por cuidados. Embora a análise ecológica não permita inferir o comportamento individual, a coocorrência de altas taxas de escolaridade e alta CV nos mesmos territórios corrobora estudos que apontam a educação materna como fator robusto para melhores desfechos de saúde infantil^40–46^. Uma meta-análise global demonstrou que a chance de vacinação completa é mais que o dobro entre filhos de mães com ensino secundário ou superior, embora haja variação do efeito conforme o contexto socioeconômico e geográfico^47^. A atenuação dessa associação pode ser atribuída a um declínio sistêmico na CV que afetou todos os estratos sociais, reduzindo o diferencial entre os grupos educacionais.

Os padrões espaciais identificados têm implicações diretas para a vigilância em saúde, e áreas de baixa cobertura representam um risco iminente para o ressurgimento de doenças imunopreveníveis que já foram eliminadas no país. A análise em escala municipal, como a realizada neste trabalho, é, portanto, uma ferramenta estratégica para o planejamento de ações de saúde pública, permitindo que gestores direcionem recursos e campanhas de intensificação vacinal para territórios de maior risco.

Os agrupamentos não são aleatórios, mas sim a manifestação espacial das profundas e persistentes desigualdades socioeconômicas e estruturais que caracterizam a região Norte. Historicamente, a região enfrenta desafios de um sistema de saúde frágil, infraestrutura precária e barreiras de acesso, especialmente para suas vastas e frequentemente isoladas populações, como comunidades indígenas, ribeirinhas e quilombolas. A pandemia de COVID-19 não originou essas disparidades, mas as aprofundou e expôs de forma notável, criando um cenário de risco epidemiológico que demanda ações urgentes, territorialmente focadas e culturalmente sensíveis.

Este estudo pode conter limitações inerentes ao seu delineamento ecológico, o que impede a inferência de causalidade em nível individual. Além disso, a utilização de dados secundários do DATASUS está sujeita a possíveis vieses e inconsistências no registro das informações, como taxas de cobertura artificialmente infladas (acima de 100%) ou subestimadas. CVs acima de 100% podem estar relacionadas ao fato de o numerador se basear no local de aplicação da vacina e não no de residência do vacinado, bem como às estimativas populacionais desatualizadas no denominador. Neste estudo, optou-se por manter os valores originais registrados, sem truncamento, visando preservar a variabilidade estatística necessária para a detecção sensível de *clusters* e evitar o viés de homogeneização, ou “efeito teto”, que ocorreria ao igualar municípios com desempenhos distintos. Essa abordagem permite ainda refletir o volume real de doses aplicadas e capturar fenômenos espaciais genuínos, como a polarização de atendimento em municípios vizinhos e o efeito de borda.

Contudo, a robustez da análise espacial e a significância estatística dos padrões encontrados conferem validade aos nossos achados, reforçam a urgência do problema e fornecem subsídios para o planejamento e direcionamento estratégico de ações em saúde pública para áreas de maior risco.
